# A Phase IIa clinical trial to evaluate the effects of anti-retroviral therapy in Alzheimer’s disease (ART-AD)

**DOI:** 10.1038/s44400-024-00001-z

**Published:** 2025-03-12

**Authors:** A. Campbell Sullivan, Gabrielle Zuniga, Paulino Ramirez, Roman Fernandez, Chen-Pin Wang, Ji Li, Lisa Davila, Kristine Pelton, Sandra Gomez, Claira Sohn, Elias Gonzalez, Marisa Lopez-Cruzan, David A. Gonzalez, Alicia Parker, Eduardo Zilli, Gabriel A. de Erausquin, Sudha Seshadri, Sara Espinoza, Nicolas Musi, Bess Frost

**Affiliations:** 1 Glenn Biggs Institute for Alzheimer’s and Neurodegenerative Diseases, University of Texas Health San Antonio, San Antonio, TX USA; 2 Department of Neurology, University of Texas Health San Antonio, San Antonio, TX USA; 3 Barshop Institute for Longevity and Aging Studies, University of Texas Health San Antonio, San Antonio, TX USA; 4 Department of Cell Systems and Anatomy, University of Texas Health San Antonio, San Antonio, TX USA; 5 Brown University Center for Alzheimer’s Disease Research, Providence, RI USA; 6 Department of Population Health Sciences, University of Texas Health San Antonio, San Antonio, TX USA; 7 Department of Medicine, Cedars-Sinai Medical Center, Los Angeles, CA USA; 8 Department of Psychiatry and Behavioral Sciences, University of Texas Health San Antonio, Department of Neurological Sciences, San Antonio, TX USA; 9 Rush University Medical Center, Chicago, IL USA

**Keywords:** Alzheimer's disease, Clinical trials

## Abstract

Retrotransposons constitute over 40% of the human genome. Studies in *Drosophila*, mice, cultured cells, and human brain show that retrotransposons are activated in tauopathies, including Alzheimer’s disease, and causally drive neurodegeneration. The reverse transcriptase inhibitor 3TC (lamivudine) reduces retrotransposon activation and suppresses tau neurotoxicity among model systems. This phase 2a open-label trial (Pilot Study to Investigate the Safety and Feasibility of Anti-Retroviral Therapy for Alzheimer’s Disease, NCT04552795, registered 09/10/2020) followed 12 participants with early Alzheimer’s disease (MMSE > 24, CDR = 0.5) over 24 weeks to assess safety, tolerability, and feasibility of daily 300 mg 3TC treatment. The sample was well-educated (12-20 years) and culturally diverse (25% from underrepresented groups). In addition to a favorable safety profile and stable cognitive measures, notable significant changes in fluid-based biomarkers include reduction of glial fibrillary acidic protein (GFAP) (*P* = 0.03) in CSF, suggestive of reduced neuroinflammation, and elevation of Aβ42/40 (*P* = 0.009) in plasma, suggestive of reduced plaque load in the brain. These results warrant further exploration in a larger, placebo-controlled trial.

## Introduction

While the estimated global prevalence of Alzheimer’s disease dementia is frequently cited as greater than 50 million people^1^ and costs over a trillion dollars annually in the United States, these estimates fail to capture those in the early stages of the disease process. When looking across the Alzheimer’s disease continuum, from those in the ‘preclinical’ stages to those with advanced dementia, the global estimates balloon to approximately 416 million people^2^, accounting for roughly 22% of individuals over the age of 50 worldwide.

Given the high prevalence rates and mounting economic and societal burdens, efforts to develop disease-modifying treatments for Alzheimer’s disease have accelerated. The FDA recently approved two treatments targeting the removal of abnormal aggregates of Aβ in the brain. While promising, these anti-amyloid treatments have yielded limited clinical results in patients with Alzheimer’s disease^3^. Similar lackluster findings have been reported for efforts targeting tau removal in the brain^4,5^. While strategies to clear aggregated Aβ and tau remain promising, these proteins begin to accumulate in the brain decades prior to a clinical diagnosis of Alzheimer’s disease^6^. The nodes within the sequela of toxic events induced by Aβ or tau deposition thus provide novel pharmacological targets for mitigating disease progression.

We^7–9^ and others^10–12^ have reported that pathogenic forms of tau drive neurotoxicity through the activation of retrotransposons in *Drosophila*, cell, and mouse models of tau pathology. Retrotransposons are “selfish” genetic elements that compose over 40% of the human genome. Such elements are thought to have derived from viral infections that occurred over the course of evolution; intact retrotransposons are similar to retroviruses in that they have the ability to replicate their DNA via an RNA intermediate and insert the new copy into the genome^13^. In addition to generating insertional mutations, retrotransposons can activate the innate immune system through the production of viral-like double stranded RNAs (dsRNA), extrachromosomal circular DNAs (eccDNA), and proteins^14–19^, and can cause DNA double-strand breaks as a consequence of failed insertion^20^. Transcriptional analyses of post-mortem human brain identify retrotransposon-encoded transcripts, particularly of the “endogenous retrovirus” class, that are elevated in Alzheimer’s disease and progressive supranuclear palsy, a “primary” tauopathy^7^, and suggest that such activation is a consequence of pathogenic forms of tau^10^. In line with these findings, longitudinal analysis of blood isolated from patients with Alzheimer’s disease further reveals a significant increase in retrotransposon transcripts prior to phenoconversion from normal cognition to cognitive impairment^21^. Work in model organisms of tauopathy demonstrates that retrotransposon activation is a causal factor driving neurodegeneration and neuroinflammation^7,9–12^.

The reverse transcriptase inhibitor 3TC effectively reduces retrotransposon activation and neurotoxicity in *Drosophila*^7^, mouse^11,12^, cell^11^, and brain spheroid^22^ models of tauopathy and Alzheimer’s disease. 3TC is an anti-retroviral medication approved by the FDA in the 1990’s to treat HIV/AIDS and chronic hepatitis B and continues to be prescribed as a mono- or combination therapy for retroviral infection. 3TC is considered safe, is widely prescribed, and has few clinically-relevant pharmacological interactions due to its low metabolic clearance, minimal binding to plasma protein, and no detectable effects on liver function^23–26^. In addition to the positive effects of 3TC in laboratory models of Alzheimer’s disease and related tauopathies, analysis of health insurance databases in the United States indicates that the use of nucleoside reverse transcriptase inhibitors such as 3TC is associated with lower incidence of Alzheimer’s disease^27^. Given this compelling preclinical data and the encouraging safety profile of 3TC, we conducted the first clinical trial of 3TC in older adults with predementia Alzheimer’s disease.

## Results

### Study design, participant characteristics, and drug adherence

The primary outcomes of this 24-week open-label phase 2a trial were to evaluate the feasibility, CNS penetration, effects on circulating levels of reverse transcriptase activity, and safety and tolerability of the nucleoside analog reverse transcriptase inhibitor 3TC in subjects with mild cognitive impairment due to suspected Alzheimer’s disease. Secondary outcomes explored cognitive performance and changes in CSF and plasma biomarkers related to neurodegeneration and neuroinflammation from baseline to post-treatment. This study was launched during COVID. As such, we opted for a single-arm and targeted approach in order to meet recruitment goals and reduce unnecessary exposure. 22 candidates were pre-screened to identify 12 participants aged 50-99 years with a Clinical Dementia Rating (CDR) of 0.5 and Mini-Mental State Examination (MMSE) score between 24 and 30. Eligibility criteria are included in Supplementary Table 1. Participants were enrolled between March 18, 2021 and September 15, 2022. Baseline participant characteristics are summarized in Table 1.

**Table 1:** Study participant characteristics

CHARACTERISTIC	*N*
Number of participants	12
Average age, years (min, max)	69.4 (52, 83)
Age group (years)	
≤65	3 (25%)
65–74	5 (41.7%)
75–84	4 (33.3%)
Sex	
Female	9 (75%)
Male	3 (25%)
Race	
White, non-Hispanic	9 (75%)
White, Hispanic	1 (8.3%)
African American	1 (8.3%)
Asian	1 (8.3%)
Education, years (SD)	15.3 (2.4)
Past medical history	
≤1 Comorbidities	2 (16.7%)
2+ Comorbidities	10 (83.3%)
Dyslipidemia	9 (75%)
Prediabetes	4 (33.3%)
Diabetes	2 (16.7%)
Hypertension	6 (50%)
Duration of symptoms prior to trial	
≤1 year	1 (8.3%)
1–2 years	2 (16.7%)
3+ years	2 (16.7%)
Unknown	7 (58.3%)
Average BMI, kg/m^2^ (SD)	25.2 (4.6)
Concurrent treatment with donepezil	7 (58.3%)

Following the initial screening visit, participants were subject to a comprehensive neuropsychological exam (Supplementary Table 2), blood draw (visit 1), and lumbar puncture (visit 2) prior to initiation of 300 mg daily oral 3TC (visit 3) (Table 2). Participants returned to the clinic at weeks 8, 16, and 24 of treatment (visits 4-6) to complete medication checks, physical examinations, and blood draw. At week 24 of treatment, participants completed a post-treatment comprehensive neuropsychological exam, blood draw (visit 6), and lumbar puncture (visit 7). One month after the final dose of medication, participants returned to the clinic for a final safety assessment and disenrollment (visit 8). All study participants completed the trial. CSF was not collected from three of the 12 participants due to procedure failure, procedure-related pain, or patient-reported illness.

**Table 2:** Study visit schedule

	Screening	Visit 1	Visit 2	Visit 3	Visit 4	Visit 5	Visit 6	Visit 7	Visit 8
	Safety measurements								
Vitals/labs	X	X		X	X	X	X		X
Height/weight	X	X		X			X		
Adverse event review				X	X	X	X		X
	Neuropsychological testing								
CDR, MMSE	X								
Neuropsychological battery		X					X		
	Clinical procedures								
Blood draw	X	X			X	X	X		
Lumbar puncture			X					X	
	Study medication								
Administer in clinic				X	X	X			
Compliance/tolerability					X	X	X		

3TC is absorbed rapidly after oral administration, reaching peak serum concentrations within 1.5–3 hours^28^. Previous analysis of 3TC brain penetration reports that the concentration of 3TC in CSF is 4–8% of serum concentrations 2–4 hours following treatment. Blood draws and lumbar punctures for 3TC analyses were performed within 24 hours of drug administration. 3TC was detected in plasma and CSF in all participants at all time points (Supplementary Figs. 1a, b), indicative of brain penetration and in line with full medication adherence as reported by all participants and caregivers at each visit. Plasma and CSF 3TC levels varied widely among participants and study visits, likely due to the short half-life of 3TC.

Some retrotransposons encode a viral-like reverse transcriptase that acts within the cell to convert retrotransposon RNA into new DNA copies. While establishing target engagement of 3TC with a retrovirus is straightforward, there is, no established assay to quantify 3TC target engagement with retrotransposons in humans. We nevertheless quantified reverse transcriptase activity in plasma collected from participants at baseline and after 24 weeks of 3TC treatment using a modified version of the EnzCheck Reverse Transcriptase Assay, a fluorescent dye-based assay that quantifies the ability of a given sample to generate RNA:DNA heteroduplexes from a poly(A) template^29^. Levels of reverse transcriptase activity in plasma at baseline and following 24 weeks of 3TC treatment were variable among participants and did not significantly differ in response to treatment (Supplementary Fig. 2). Reverse transcriptase activity was not detected in CSF.

### Safety and treatment-emergent adverse events

3TC is widely used in antiretroviral therapy regimens due to its potent and long-lasting antiviral efficacy and low toxicity. Adverse events defined as unfavorable medical occurrences temporally associated with the subject’s participation in the research were reviewed at visits 3-6 and at the follow-up visit one month following the last administration of 3TC. There was one event of gastrointestinal bleeding due to a peptic ulcer in a participant who was taking daily aspirin. There was one report of mild fatigue, and one report of mild muscle pain. Two subjects reported mild headache after lumbar puncture. Two participants reported a COVID-19 infection during the treatment period. Serial laboratory studies did not reveal signs of liver toxicity (e.g. elevated liver enzymes, bilirubin, hypoglycemia, anemia) or cytopenia, which are previously-reported 3TC-related adverse events^30–32^. Given that weight loss has been reported in previous studies of 3TC-containing antiretroviral regimens^33^, we compared standing weights at baseline and after 24 weeks of treatment. Subjects lost 1.83 kg on average, ranging from 8.62 kg loss to 3.99 kg gain, after daily 3TC for 24 weeks. No deaths occurred within the treatment period or one month after treatment.

### Effects of 3TC on cognition and functional status

Following 24 weeks of treatment, we measured cognitive change from baseline using a comprehensive neuropsychological battery (Table 3) as an exploratory outcome. There was no significant change in cognition among participants from baseline to follow-up assessment. In addition to exploring post-treatment changes in cognition across the test battery, secondary aims assessed changes in the Preclinical Alzheimer Cognitive Composite (PACC-5) score. Although typically relegated to individuals with prodromal and asymptomatic disease, the PACC-5 was included given its sensitivity to Alzheimer’s disease-specific cognitive change^34^. Similarly, no statistically significant changes were observed. While we detect a trend for small decreases in MMSE (*P* = 0.06) and PACC-5 (*P* = 0.07) with treatment, this trend resolves after participants with a COVID-19 infection during the treatment period are removed from the analysis (MMSE, *P* = 0.2; PACC-5, *P* = 0.2). MMSE and PACC-5 scores for each participant are included in Supplementary Fig. 3. Overall, these findings are reassuring and suggest cognitive stability over the six-month period.

**Table 3:** Cognitive assessments

DOMAIN	TEST	BASELINE MEDIAN (IQR)	POST-TREATMENT MEDIAN (IQR)	*P*-value
Global	MMSE	27 (25, 29)	25 (23, 28)	0.06
AD composite	PACC-5 (Z-score)	−1.59 (−2, −0.59)	−1.59 (−2, −1.2)	0.07
Attention & working memory	Digits Forward	6 (5, 7)	6 (5, 7)	0.8
	Digits Backward	4 (4, 5)	4 (4, 5)	0.2
Verbal list learning	HVLT Learning	15 (14, 18)	16 (13, 21)	>0.9
	HVLT Recall	0 (0, 4)	0 (0, 4)	>0.9
	HVLT Disc	6 (2, 9)	6 (3, 9)	>0.9
Visual learning	BVMT Learning	9 (5, 11)	8.5 (6, 10)	0.8
	BVMT Recall	2 (0, 3)	2 (1, 3)	0.8
	BVMT Disc	4 (3, 5)	4 (3, 5)	0.4
Story memory	Logical Memory I	22 (18, 25)	18 (16, 24)	0.3
	Logical Memory II	6 (2, 9)	4 (0, 8)	0.8
	Logical Memory Rec	16 (14, 18)	16 (14, 16)	0.3
Episodic memory	FCSRT Delay Free	2 (0, 6)	2 (0, 6)	0.7
	FCSRT Delay Cued	9 (6, 10)	10 (8, 11)	0.8
Naming	BNT	29 (28, 29)	29 (27, 30)	0.4
Verbal fluency	Animal Naming	14 (13, 17)	15 (13, 17)	0.4
	Supermarket	20 (16, 26)	19 (16, 22)	0.11
	S Words	11 (10, 14)	11 (10, 13)	0.7
	P Words	12 (9, 13)	12 (10, 14)	0.08
Executive functioning	Trails A Time	44 (38, 64)	49 (32, 56)	0.7
	Trails B Time	114 (94, 205)	105 (92, 280)	0.4
	Digit-Symbol Coding	46 (34, 49)	39 (37, 47)	>0.9

*IQR* interquartile range. *P* value is based on Wilcoxon signed rank test with continuity correction. *N* = 12.

### Effects of 3TC on neurodegeneration-related biomarkers

**Fig. 1:**
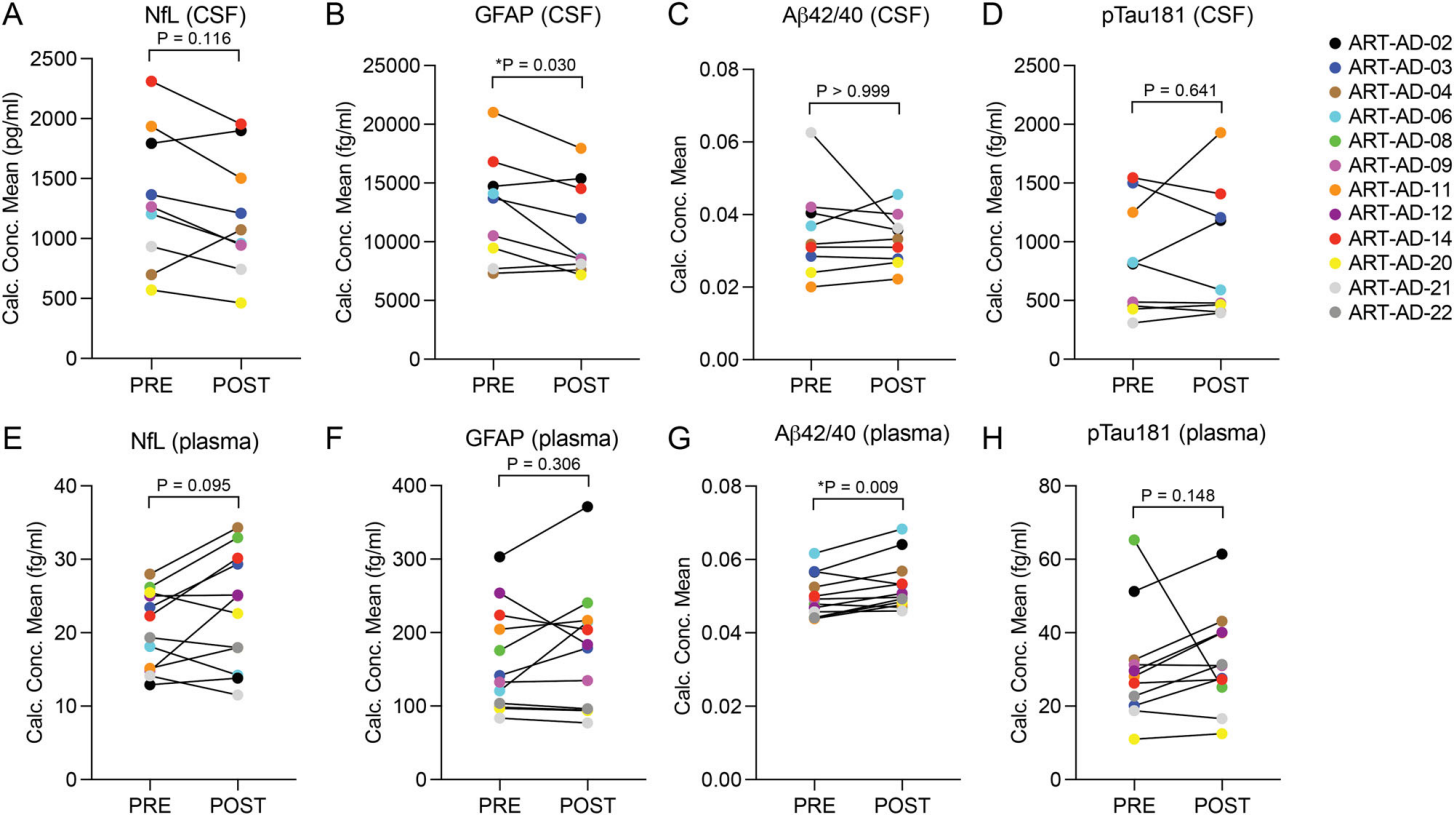
Quantification of biomarkers associated with neurodegeneration in CSF and plasma. Quanterix was used to detect the indicated proteins in CSF (**A**–**D**) and plasma (**E**–**H**) of participants at baseline and after 24 weeks of 3TC treatment. CSF, *N* = 9; Plasma, *N* = 12. Normality testing was based on the POST-PRE difference for each target; *P* values are based on two-sided paired sample t-test (normal distribution) or Wilcoxon matched-pairs signed rank test (non-normal distribution).

We next analyzed the effects of 24-week 3TC treatment on biomarkers that have been previously linked to neurodegeneration^35^ as an exploratory outcome. Commercial SIMOA (Single Molecule Array) assays were performed on an HD-X platform (Quanterix) to quantify NfL, GFAP, Aβ42, Aβ40, and pTau181 in CSF (Fig. 1A–D) and plasma (Fig. 1E, F). Despite our small sample size, we find that GFAP is significantly reduced in CSF after 24 weeks of 3TC treatment, suggestive of reduced astrocyte reactivity and neuroinflammation due to 3TC. We also detect a significant increase in Aβ42/40 in plasma after 24 weeks of 3TC treatment, suggestive of decreased amyloid pathology in the brain due to 3TC.

### Effects of 3TC on biomarkers of neuroinflammation

The Mesoscale Discoveries (MSD) V-PLEX Neuroinflammation Panel 1 was used to analyze CSF and plasma levels of 37 biomarkers that are associated with a neuroinflammatory response. Biomarkers that were either not detected in more than three participants or those in which the % coefficient of variation (%CV) exceeded 15 were removed from the analysis. This left 21 high-confidence targets for analysis in CSF and 30 high-confidence targets for analysis in plasma (Table 4, Supplemenary Figs. 4, 5). In CSF, we detect a trending reduction in Flt1 from baseline to post-treatment (*P* = 0.05). In plasma, we detect an increase in IL-15 from baseline to post-treatment (*P* = 0.006). The remaining neuroinflammatory biomarkers were not significantly changed over the course of treatment.

**Table 4:** CSF and plasma-based inflammatory biomarker analysis

CSF			PLASMA		
PROTEIN	POST-PRE (IQR)	*P* VALUE	PROTEIN	POST-PRE (IQR)	*P* VALUE
bFGF	NA	NA	bFGF	0.6377 (−1.97, 1.02)	>0.9999
CRP	39.06 (-7246, 1342)	>0.9999	CRP	−903927 (−2106270, 118451)	0.063
Eotaxin	NA	NA	Eotaxin	210.8 (39.0, 439)	0.092
Eotaxin-3	NA	NA	Eotaxin-3	3.271 (−1.88, 7.34)	0.398
Flt1	−6.977 (−14.0, 1.15)	0.050	Flt-1	−1.081 (−7.16, 1.57)	0.380
IFN-g	NA	NA	IFN-g	0.9857 (-0.233, 1.46)	0.206
IL-1a	NA	NA	IL-1a	NA	NA
IL-1b	NA	NA	IL-1b	NA	NA
IL-2	NA	NA	IL-2	NA	NA
IL-4	NA	NA	IL-4	NA	ND
IL-5	−0.03144 (-0.119, 0.454)	0.298	IL-5	−0.01947 (−0.169, 0.0651)	0.718
IL-6	−0.06009 (−0.0209, 0.0595)	0.364	IL-6	−0.04436 (−0.866, 0.275)	0.622
IL-7	−0.01858 (−0.0544, 0.0745)	0.576	IL-7	0.1686 (−1.61, 0.441)	0.311
IL-8	−1.844 (−5.63, 2.45)	0.377	IL-8	0.5924 (−1.21, 17.9)	0.677
IL-10	NA	NA	IL-10	0.04822 (−0.0142, 0.0854)	0.064
IL-12_			IL-12_		
IL-23p40	0.1495 (−0.576, 0.459)	>0.9999	IL23p40	−0.5283 (−34.3, 31.2)	0.967
IL-13	NA	NA	IL-13	NA	NA
I-15	0.09699 (−0.284, 0.345)	0.652	IL-15	0.2861 (0.150, 0.456)	***0.006
IL-16	0.1714 (−0.284, 0.449)	0.719	IL-16	0.9511 (−10.1, 10.6)	0.860
IL-17a	−0.002132 (−0.0867, 0.0664)	0.956	IL-17A	−0.04166 (−0.141, 0.0588)	0.417
IP-10	12.55 (−82.4, 40.3)	0.910	IP-10	30.29 (−46.4, 61.2)	0.470
MCP-1	21.2 (5.33, 34.7)	0.076	MCP1	15.5 (−18.1, 52.1)	0.208
MCP-4	−0.006317 (−0.0945, 0.140)	0.884	MCP4	47.17 (−31.7, 82.5)	0.155
MDC	NA	NA	MDC	−14.69 (−44.7, 86.7)	>0.9999
MIP-1a	NA	NA	MIP-1a	1.421 (−0.391, 3.30)	0.279
MIP-1b	−0.6947 (−2.51, 1.42)	0.383	MIP-1b	−0.2869 (−18.2, 8.90)	0.6772
PIGF	−0.7235 (−1.71, 0.180)	0.118	PIGF	−0.05237 (−0.919, 0.942)	0.883
SAA	−72.3 (−394, 4.49)	0.055	SAA	−109699 (−1116509, 173190)	0.340
siCAM	219.8 (−112, 480)	0.165	siCAM	−10037 (−67421, 46858)	0.605
svCAM	−57.57 (−647, 638)	0.857	svCAM	8605 (−46964, 50495)	0.721
TARC	−0.157 (−0.509, 0.121)	0.257	TARC	3.749 (−12.8, 10.4)	0.850
Tie-2	NA	NA	Tie-2	−123.3 (−623, 257)	0.331
TNF-a	NA	NA	TNF-a	0.006858 (−0.102, 0.201)	0.791
TNF-b	NA	NA	TNF-b	NA	NA
VEGF-A	0.08938 (−0.0830, 0.355)	0.203	VEGF-A	0.1319 (−1.01, 1.36)	0.850
VEGF-C	−8.791 (−28.8, 7.79)	0.192	VEGF-C	2.184 (−70.6, 49.5)	0.791
VEGF-D	−3.987 (−9.22, 3.48)	0.199	VEGF-D	81.78 (−88.2, 245)	0.250

A value of “NA” is included for targets in which three or more participants had undetectable values or %CV values exceeding 15. POST-PRE values are in pg/ml. CSF, *N* = 9; Plasma, *N* = 12. Normality testing was based on the POST-PRE difference for each target; *P* values are based on two-sided paired sample t-test (normal distribution) or Wilcoxon matched-pairs signed rank test (non-normal distribution). *IQR* Interquartile Range.

## Methods

### Study design and intervention

ART-AD is an investigator-initiated, single-center, open-label phase 2a clinical trial. Enrolled subjects were stable on current medications for at least eight weeks prior to initiating 300 mg oral 3TC (McKesson Medical Supplies, product #1121405) daily at the initial visit. This dose was maintained throughout the 24-week study with excellent medication adherence as assessed by pill count. All 12 subjects received the same dose and frequency. Subjects presented to the clinic at regular visits according to the protocol outlined in Table 2. This study was conducted according to US and international standards of Good Clinical Practice (FDA Title 21 part 312). All participants provided written informed consent. Participant identification numbers were not known to any individuals outside of the research group. The study protocol was approved by the University of Texas Health San Antonio Institutional Review Board and was registered at ClinicalTrials.gov (NCT04552795) on 09/10/2020 as “Pilot Study to Investigate the Safety and Feasibility of AntiRetroviral Therapy for Alzheimer’s Disease.”

### Fluid collection and processing

Fasting peripheral venous blood was collected into BD Paxgene® DNA tubes, BD EDTA-coated tubes, and BD serum separator tubes (SST) for DNA, hematology, and chemistry analyses, respectively. Peripheral blood mononuclear cells (PBMC) were isolated from plasma using BD CPT tubes that contain Ficoll-Hypaque. Plasma and PBMCs were aliquoted into polypropylene tubes and stored at -80 °C. Complete blood count with differential and platelets, coagulation, metabolic, and lipid panels, and hemoglobin A1c were analyzed by LabCorp.

Lumbar punctures were performed at the Glenn Biggs Institute for Alzheimer’s and Neurodegenerative Diseases after overnight fasting. 2 ml of CSF was collected into a 10 ml polypropylene tube via gravity drip using a spinal needle. The resulting CSF was centrifuged to remove cellular debris, then aliquoted into polypropylene tubes and stored at -80 °C.

### Neuropsychological assessment

All participants completed a comprehensive neuropsychological assessment (see Supplementary Table 2). These evaluations were completed at baseline (prior to the initiation of 3TC), and then again following 24-weeks of continued treatment. The PACC-5 score was calculated as a mean normative z-score across five measures, including MMSE, Logical Memory Delayed Recall, Digit-Symbol Coding Test, Category Fluency, and Free and Cued Selective Reminding Test.

### HPLC/MS/MS chromatography

3TC ((−)-L-2′,3′-dideoxy-3′-thiacytidine) and internal standard (3-guanidinopropionic acid (3GPA)) were obtained from Millipore Sigma. All other HPLC-grade reagents were purchased from Thermo Fisher Scientific. All solutions were prepared with Milli-Q water. A 3TC super stock was prepared in methanol at a concentration of 1 mg/ml and stored in aliquots at -80 °C. The working stock solution of 3TC was prepared each day from the super stock at a concentration of 100 μg/ml to spike the calibrators.

Reference calibrators were prepared by spiking control plasma to concentrations of 0, 5, 10, 25, 50, 100, 500, 1000 ng/ml of 3TC, and control CSF to concentrations of 0, 2, 10, 50, 100, 500, 1000, 5000 ng/ml of 3TC. An identical concentration of internal standard, 10 µl (100 µg/ml 3-GPA), was spiked into each of the calibrators and blinded samples. 100 µl of mobile phase B (0.1% formic acid in acetonitrile) was added to each tube, vortexed thoroughly, and spun for 5 minutes at 17,000 x *g*. 10 µl of the supernatant was injected into the HPLC with MS detection.

The LC/MS/MS system consisted of a Shimadzu SIL 20 A HT autosampler, two LC-20AD pumps, and an AB Sciex API 4000 tandem mass spectrometer with turbo ion spray. The LC analytical column was an ACE Excel C18-PFP (75 × 3.0 mm, 3 micron) purchased from Mac-Mod Analytical and was maintained at 24 °C during the chromatographic runs using a Shimadzu CT-20A column oven. Mobile phase A containing 0.1% formic acid dissolved in water was run at 0.255 ml/min. Mobile phase B consisted of 0.1% formic acid dissolved in 100% HPLC grade acetonitrile and run at 0.045 ml/min. The total flow rate of the mobile phase was 0.3 ml/min. 3TC and the internal standard, 3-GPA, were eluted isocratically with a 5-minute run. 3TC eluted at 3.69 min, while 3-GPA eluted at 3.25 min. Drug transitions were detected in positive mode at m/z 230 à 112 for 3TC and m/z 132 à 72 for 3-GPA. The ratio of 3TC peak area to the internal standard peak area for each blinded sample was compared against a linear regression of the ratios obtained by the calibration peak areas to quantify 3TC.

### Reverse transcriptase activity

Quantification of reverse transcriptase activity was performed using the EnzCheck reverse transcriptase assay kit (Invitrogen, E22064). Frozen plasma and CSF samples were thawed on ice and centrifuged at 2000 rpm for 10 minutes to remove debris. Protein within the resulting supernatant was concentrated using 50 K molecular weight cut-off concentrators (Thermo Fisher Scientific, 88539). Reverse transcriptase reactions were run with diluted samples in freshly prepared enzyme dilution buffer (50 mM Tris-HCl, 20% glycerol, 2 mM DTT, pH 8) to microplate wells with and without the reaction mixture containing template/primer solution in polymerization buffer. The reaction was incubated for six hours at room temperature, after which 200 mM EDTA was added to stop the reaction. RNA-DNA heteroduplexes formed in the reaction were detected via PicoGreen; fluorescence was measured using a microplate reader (Promega GloMax Discover). To correct for nonspecific binding, the fluorescence level of the sample (plasma sample with reaction mixture) was subtracted from the background (plasma sample without reaction mixture). The corrected data was used to calculate reverse transcriptase activity based on the reverse transcriptase standard curve.

### Fluid biomarker analysis

Plasma and CSF samples were analyzed in the Fluid Biomarkers Laboratory at the Brown University Center for Alzheimer’s Disease Research. Samples and controls were run in duplicate and calibrators were run in triplicate. The measurements were performed blinded to diagnosis and clinical data in one round of experiments using one batch of reagents. Biomarkers related to neurodegeneration and neuroinflammation were quantified from the same CSF or plasma sample for each subject. The mean inter-assay and intra-assay CV values were below 10% for all markers. Samples with CV values greater than 15% were removed from the analysis. Targets lacking data for more than three participants (due to lack of detection or CV > 15%) were not included in the analysis.

#### Measurement of Aβ40, Aβ42, NfL, GFAP and pTau181

Plasma and CSF levels of the analytes of interest were determined by commercial SIMOA (Single Molecule Array) assays on an HD-X platform (Quanterix). Levels of Aβ42, Aβ40, GFAP, and NfL were quantified using the Neurology 4-plex E kit (103670; Quanterix) in CSF (400-fold dilution) and plasma (4-fold dilution). Levels of pTau181 were quantified using the pTau181 Advantage kit version 2.1 (104111; Quanterix) in CSF (10-fold dilution) and plasma (4-fold dilution).

Samples were thawed briefly on wet ice. Immediately after thawing, CSF samples used for Aβ40, Aβ42, NfL, and GFAP assays were diluted 400x in N4PE CSF Sample Diluent. All calibrators, controls, and samples were then incubated at room temperature for one hour. Samples and internal controls were centrifuged at 10,000 x g for five minutes. All plasma samples were diluted 4x onboard using the provided sample diluent. CSF samples were diluted at the bench and run neat. A four-parameter logistic curve fit, 1/y2 weighted was used for GFAP, NfL, and pTau181 while a 5-paramter logistic curve fit, 1/y2 weighted was used for Aβ40 and Aβ42. Two control samples of known concentration (high-control and low-control) provided in each kit as well as two internal controls of pooled CSF and plasma were included as quality control.

#### Measurement of inflammatory markers

Inflammatory proteins were measured according to manufacturer’s instructions using the V-PLEX Neuroinflammation Panel 1 (K15210D; Meso Scale Diagnostics) on the Meso QuickPlex SQ 120 (MSD). Plasma and CSF samples were diluted 2-fold for the V-plex Pro-inflammatory panel 1 (IFN-γ, IL-1β, IL-2, IL-4, IL-6, IL-8, IL-10, IL-13, and TNF-α), Cytokine Panel 1 (1L-1α IL-5, IL-7, IL-12/23p40, IL-15, IL-16, IL-17A, TNF-β, and VEGFA) and Angiogenesis panel 1 (VEGF-C, VEGF-D, Tie-2, Flt-1, PIGF, and bFGF). Plasma and CSF samples were diluted 4-fold for the Chemokine Panel 1 (Eotaxin, MIP-1β, Eotaxin-3, TARC, IP-10, MIP-1α, MCP-1, MDC, and MCP-4). CSF samples were diluted 5-fold and plasma samples were diluted 1000-fold for the Vascular Injury Panel 1 (SAA, CRP, VCAM-1, and ICAM-1). Levels of MCP-4 (CSF), MIP-1a (plasma), TARC (CSF), IL-17a (CSF), IL-10 (plasma), IL-5 (plasma), IL-17a (plasma) fell below the lower limit of detection (LLOD) during the initial screen. The assay was repeated for those markers only using a 2-fold dilution with an overnight incubation at 4°C^36^.

Samples were thawed briefly on wet ice and centrifuged at 2000 x *g* for three minutes. After washing of the precoated plates with Phosphate Buffered Saline plus 0.05% Tween-20, 50 μl of calibrator control and the diluted sample was added to each well. Plates were sealed and incubated for two hours at room temperature or overnight at 4 °C with shaking at 700 rpm. After washing, 25 μl of detection antibody solution was added to each well and the plate was incubated for two hours at room temperature with shaking at 700 rpm for all assays except Vascular Injury Panel 1, which was incubated for one hour. The plates were washed again followed by the addition of 150 μl Read Buffer T to each well. Plates were read immediately with the exception of Chemokine Panel 1, which was incubated for ten minutes at room temperature prior to reading. A four-parameter logistic curve fit, 1/y2 weighted was used for all assays. Control samples of known concentration were included as quality control.

### Statistical analyses

#### Cognitive assessments

Given the small sample size and skewed distribution, non-parametric (Wilcoxon matched-pairs signed rank test) tests were used to examine cognitive change following 24-weeks of 3TC. SPSS was used for statistical analyses of cognitive data.

#### Fluid biomarker analyses

For each target, the range of POST-PRE values among all participants was tested for normality using D’Agostino’s K-squared test. A paired two-sided t-test was used to calculate *P* values for targets in which POST-PRE values passed the normality test (alpha = 0.05), while *P* values for targets that failed the normality test were calculated using the Wilcoxon matched-pairs signed rank test. A *P* value of less than or equal to 0.05 was considered significant. As pre-specified in our statistical plan, multiple comparison testing was not performed due to the exploratory nature of the study, small sample size, and to avoid false negatives.GraphPad Prism was used for statistical analyses and figure preparation.

## Discussion

In the current study, we provide results of the first clinical trial testing the effects of 3TC in participants with mild cognitive impairment due to suspected Alzheimer’s disease. We find that 24-week treatment with 300 mg daily oral 3TC (the standard dosing regimen for HIV and hepatitis B) is safe and well-tolerated, and that 3TC effectively penetrates the blood-brain barrier in this population. 3TC is absorbed rapidly after oral administration, with an absolute bioavailability of 82% in adults^37^. 3TC was detected in the plasma and CSF of all participants, indicating adherence to the study drug. 3TC levels were variable among participants and between study visits. The variability of 3TC detected in plasma and CSF among participants likely reflects the short half-life of 3TC. In future studies, we suggest controlled timing between dosing and fluid sampling to decrease variability among subjects and timepoints. The active anabolite of 3TC, 3TC 5’-triphosphate (3TC-TP), has a relatively longer half-life of approximately 15 hours within cells^28^. While analysis of 3TC-TP would provide a quantitative assessment of active drug in each participant, this approach requires measurements of the drug within isolated cells, which is a challenge for quantification in CSF.

While our study was not powered to assess efficacy, significant changes were detected in biomarkers related to neurodegeneration and neuroinflammation. In longitudinal studies, low levels of plasma Aβ42/40 and high levels of plasma pTau181, GFAP, and NfL are associated with subsequent cognitive decline^35^. In the current analysis, we find significant changes in fluid levels of Aβ42/40 and GFAP, and trending changes in NfL after six months of 3TC treatment. Plasma Aβ42/40 is inversely correlated with amyloid burden in the brain such that individuals with positive amyloid PET have significantly lower levels of Aβ42/40^38,39^. A lower Aβ42/40 ratio is also associated with amnestic mild cognitive impairment, and individuals with lower baseline levels of Aβ42/40 have an increased risk of progression to dementia^40,41^. We find a significant elevation of Aβ42/40 in plasma after six months of 3TC treatment compared to baseline. Elevated plasma Aβ42/40 suggests a potentially protective effect of 3TC on the amyloid accumulation in the brain, in line with recent work reporting reduced Aβ deposits in induced neurons from patients with late-onset Alzheimer’s disease in response to 3TC exposure^42^. While levels of Aβ42/40 in CSF were unchanged after six months of 3TC treatment in the current study, such discordance between plasma and CSF Aβ42/40 is perhaps not unexpected, as a previous analysis leveraging the BioFINDER and Alzheimer’s Disease Neuroimaging Initiative cohorts reports a moderate Spearman correlation coefficient of 0.31 between plasma and CSF Aβ42/40 in individuals with Alzheimer’s disease based on the Simoa N4PE assay^43^, which was used in the current study.

We detect a significant reduction of GFAP in CSF after six months of 3TC treatment. GFAP is a marker of reactive astrogliosis that is elevated in plasma and CSF of patients with preclinical, prodromal, and Alzheimer’s disease dementia^35,40,44–46^. While the trending decrease in CSF levels of NfL, a marker of axonal damage that is elevated in CSF^47^ and plasma^48^ of individuals with mild cognitive impairment, Alzheimer’s dementia, and other neurodegenerative disorders^49^, further suggests a benefit due to 3TC treatment, we note that the trending change in NfL due to 3TC treatment is discordant between CSF and plasma. Among neuroinflammatory biomarkers, we detect trending reduction of CSF Flt-1, also known as vascular endothelial growth factor receptor 1, and significant elevation of the proinflammatory cytokine IL-15 in plasma. Longitudinal biomarker analyses indicate that Flt-1 and IL-15 are elevated within CSF at preclinical, prodromal, and dementia stages of Alzheimer’s disease, and are associated with cortical thinning and subsequent cognitive decline^36^. While analyses of plasma levels of IL-15 in individuals with Alzheimer’s disease are limited, a smaller study of 20 subjects with Alzheimer’s disease compared to 15 controls reports that IL-15 is slightly but significantly reduced in the plasma of individuals with Alzheimer’s disease^50^; another study of 52 subjects with Alzheimer’s disease compared to 18 controls found no difference in IL-15 in CSF or plasma^51^. Taken together, our fluid biomarker analysis points toward compelling potential benefits of 3TC in individuals with mild cognitive impairment due to suspected Alzheimer’s disease.

We find that reverse transcriptase activity in plasma is highly variable among participants and is unchanged due to 3TC treatment. However, analysis of circulating levels of reverse transcriptase has several caveats. First, as the EnzCheck assay was performed on cell-free plasma, it is likely that levels of secreted reverse transcriptase do not reflect the level of reverse transcriptase activity within cells in circulation or within cells of the brain. Second, any type of double-stranded nucleic acid, whether it is produced via reverse transcription or another mechanism, will be detected by the assay. While analysis of retrotransposon DNA copy number could potentially be leveraged in future studies of 3TC in Alzheimer’s disease and related tauopathies, the normalization of retrotransposon DNA copies, which are highly abundant and repetitive within the human genome, to a single-copy control gene using quantitative or digital PCR-based methods is a technical caveat of this approach.

Recently-completed and ongoing studies are investigating the therapeutic efficacy of nucleoside analog reverse transcriptase inhibitors to suppress retrotransposon activation in primary and secondary tauopathies. Study results from a phase 2 trial in subjects with progressive supranuclear palsy (PSP) led by Transposon Therapeutics report a reduction in NfL, IL-6, and osteopontin after treatment with TPN-101, as well as stabilization of symptoms (NCT04993768); the FDA has granted Fast Track designation for TPN-101 for PSP based on study results. LINE-AD, a phase 1 study in which emtricitabine is being tested in participants with mild cognitive impairment or Alzheimer’s disease (NCT04500847), is ongoing.

There are several limitations in our study. First, our small sample size and lack of control group complicate interpretation and significance of biomarker findings. Further, predetermined decisions to avoid correcting for multiple comparisons given the small sample size raise concern. The lack of standardized methods to quantify 3TC target engagement with retrotransponsons in humans hindered our ability to meet primary goals.

In summary, we present findings from the first clinical trial testing the effects of reverse transcriptase inhibition in participants with mild cognitive impairment due to suspected Alzheimer’s disease. We find that 3TC is safe and well-tolerated among participants, and gains access to the CNS. We further detect beneficial effects of 3TC treatment on biomarkers associated with neurodegeneration and neuroinflammation, strongly supporting the conduct of a larger, randomized, double-blinded, placebo-controlled study of 3TC in older adults with Alzheimer’s disease and, potentially, primary tauopathies.

## Supplementary information

Supplementary information

## Data Availability

Data is provided within the manuscript and can be accessed through clinicaltrials.gov: https://clinicaltrials.gov/study/NCT04552795.
