# Supplementary material for: A Phase IIa clinical trial to evaluate the effects of anti-retroviral therapy in Alzheimer’s disease (ART-AD)
Source: NPJ Dement. 2025 Mar 12;1(1):2. doi: 10.1038/s44400-024-00001-z (PMC11917871; doi:10.1038/s44400-024-00001-z)
Supplement: Supplementary file 1 — Supplementary information [file 44400_2024_1_MOESM1_ESM.pdf]

Supplementary Information

Supplementary Figure 1 | Levels of 3TC in plasma and CSF

Supplementary Figure 2 | Reverse transcriptase activity in plasma

Supplementary Figure 3 | Difference among participants in PACC-5 z-score from baseline to post-treatment

Supplementary Figure 4 | Neuroinflammatory biomarkers in CSF

Supplementary Figure 5 | Neuroinflammatory biomarkers in plasma

Supplementary Table 1 | Eligibility criteria

Supplementary Table 2 | Neuropsychological battery

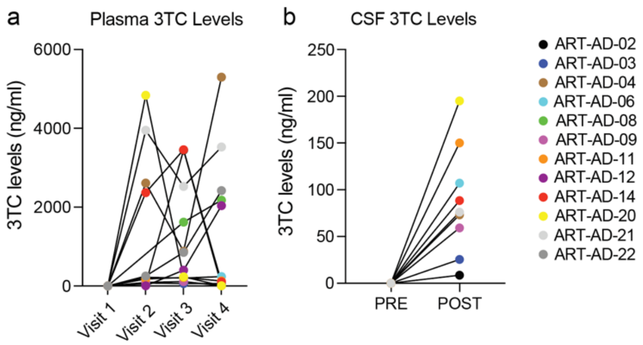

Supplementary Figure 1 | Levels of 3TC based on HPLC/MS/MS. a) Plasma, N=12, b) CSF, N=9.

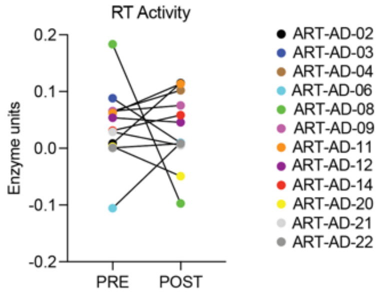

Supplementary Figure 2 | Reverse transcriptase activity in plasma. Reverse transcriptase activity was quantified using the EnzCheck Reverse Transcriptase Activity assay. N=12.

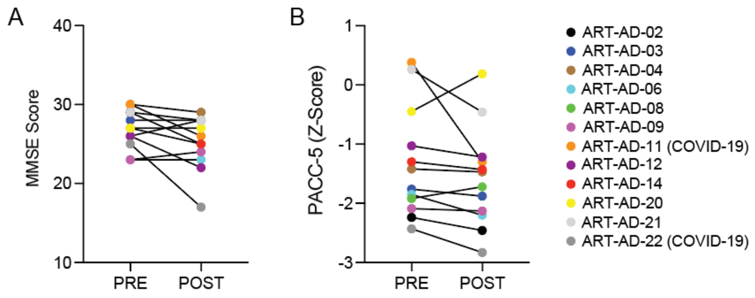

Supplementary Figure 3 | Cognitive assessment scores. a) MMSE score and b) PACC-5 Z-Scores for each participant at baseline and after 24 weeks of 3TC treatment. N=12.

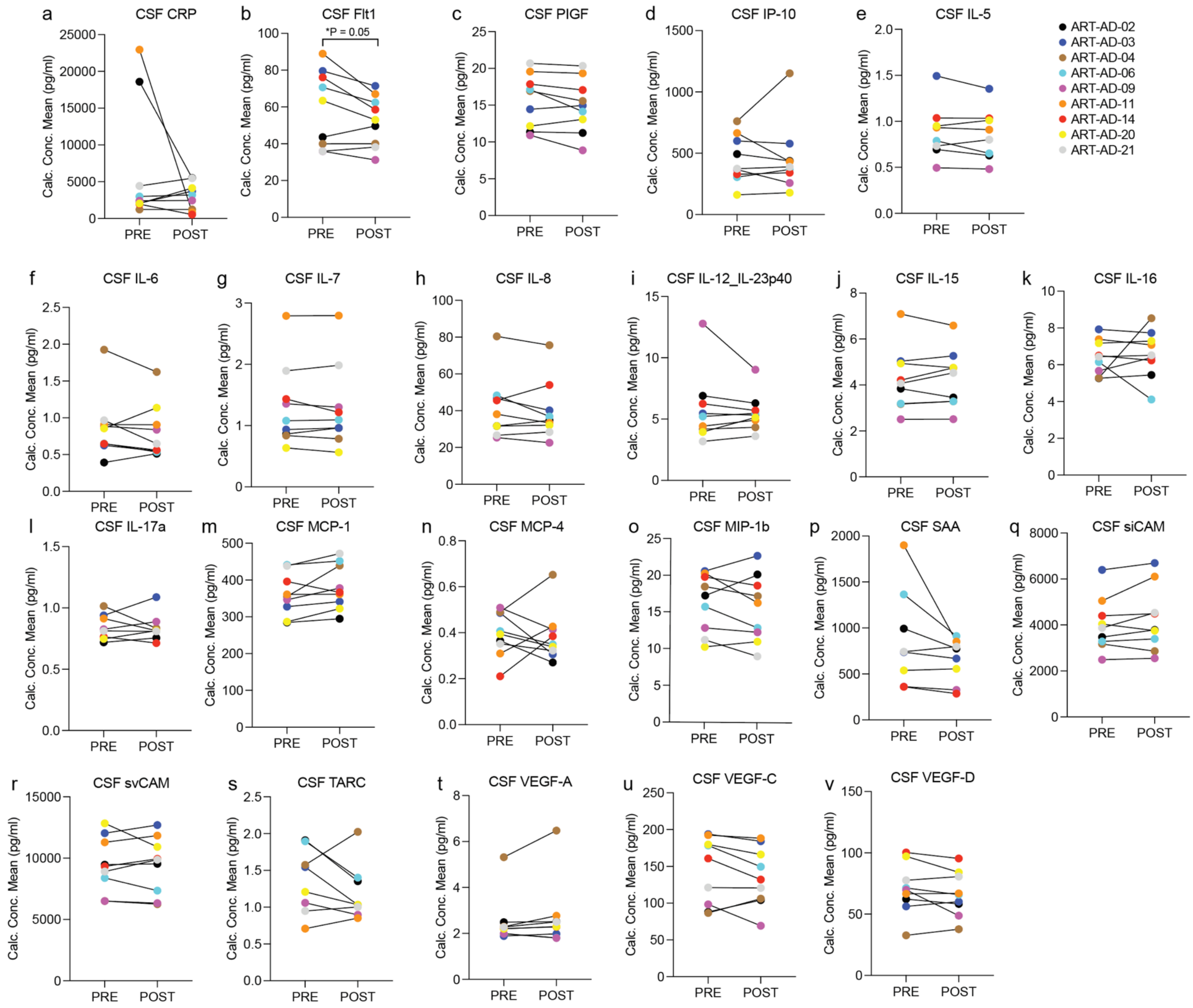

Supplementary Figure 4 | Neuroinflammatory biomarkers in CSF. The MSD platform was used to detect the indicated proteins in CSF of participants at baseline (PRE) and after 24 weeks of 3TC treatment (POST). Normality testing was based on the POST-PRE difference for each target. N=9. Significant *P* value is noted; all other *P* values are provided in **Table 4**. *P* values are based on two-sided paired sample t-test (normal distribution) or Wilcoxon matched-pairs signed rank test (non-normal distribution).

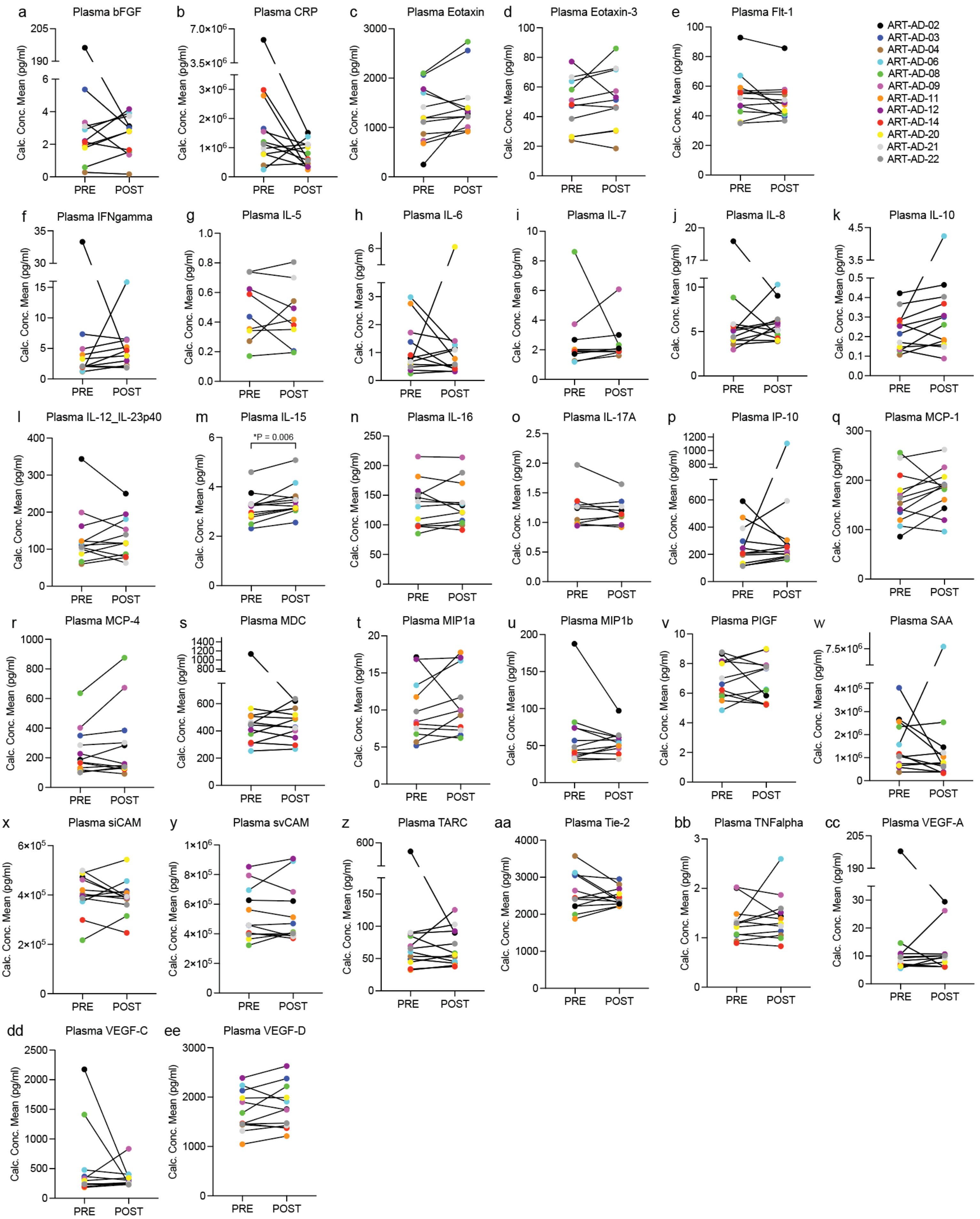

a Plasma bFGF
b Plasma CRP
c Plasma Eotaxin
d Plasma Eotaxin-3
e Plasma Flt-1
ART-AD-02
ART-AD-03
ART-AD-04
ART-AD-06
ART-AD-08
ART-AD-09
ART-AD-11
ART-AD-12
ART-AD-14
ART-AD-20
ART-AD-21
ART-AD-22
f Plasma IFNgamma
g Plasma IL-5
h Plasma IL-6
i Plasma IL-7
j Plasma IL-8
k Plasma IL-10
l Plasma IL-12_IL-23p40
m Plasma IL-15
*P = 0.006
n Plasma IL-16
o Plasma IL-17A
p Plasma IP-10
q Plasma MCP-1
r Plasma MCP-4
s Plasma MDC
t Plasma MIP1a
u Plasma MIP1b
v Plasma PIGF
w Plasma SAA
x Plasma siCAM
y Plasma svCAM
z Plasma TARC
aa Plasma Tie-2
bb Plasma TNFalpha
cc Plasma VEGF-A
dd Plasma VEGF-C
ee Plasma VEGF-D
Calc. Conc. Mean (pg/ml)
PRE
POST

Supplementary Figure 5 | Neuroinflammatory biomarkers in plasma. The MSD platform was used to detect the indicated proteins in plasma of participants at baseline (PRE) and after 24 weeks of 3TC treatment (POST). Normality testing was based on the POST-PRE difference for each target. N=12. Significant *P* value is noted; all other *P* values are provided in **Table 4**. *P* values are based on two-sided paired sample t-test (normal distribution) or Wilcoxon matched-pairs signed rank test (non-normal distribution).

INCLUSION CRITERIA
Aged 50-99 years
Clinical diagnosis of early Alzheimer's disease (Clinical Dementia Rating (CDR) = 0.5, Mini-Mental State Exam (MMSE) = 24 - 30)
If using drugs to treat symptoms related to Alzheimer's disease, doses must be stable for at least eight weeks prior to screening visit 1
Labs:<br>Adequate blood cell counts (white blood cells: 4,000 - 11,000 cells/mcL; absolute neutrophil count: 1,800-8,700 cells/mcL; platelets: 120 - 500 K/µL; hemoglobin 12.0 - 17.5 grams/dL)<br>LFTs within 2x normal value<br>CrCl ≥ 50 mL/min<br>Cholesterol (≤ 260 mg/dl), triglycerides ≤ 400 mg/dl)<br>Glucose control (HbA1c ≤ 8%)<br>Prothrombin time/partial thromboplastin time/international normalized ratio (PT/PTT/INR) within normal limits
Body mass index (BMI) within 16 – 35 kg/m^2
Reliable informant or caregiver
**EXCLUSION CRITERIA**
Medical or neurologic condition other than Alzheimer's disease that may contribute to cognitive impairment
Clinically significant unstable psychiatric illness in the past six months
Hearing, vision, or motor deficits that interfere with participation
Alcohol or drug abuse/dependence in the past six months
Stroke, transient ischemic attack, or unexplained loss of consciousness in the past six months
Unstable angina, myocardial infarction, advanced chronic heart failure, or clinically significant conduction abnormalities within the past six months
Relevant brain hemorrhage, bleeding disorder and cerebrovascular abnormalities
Diagnosis of HIV infection or AIDS (CD4 count < 200), HIV/HBV co-infection, HBV or human T-cell leukemia virus infection
History of impaired renal or liver function
Current use of memantine or sorbitol-containing products (participants who are taken off memantine may be eligible for enrollment following a one-month washout period)
HIV, HBV, or current/previous use of NRTIs/non-NRTIs
Poorly controlled blood pressure (systolic > 160, diastolic > 90 mmHg)
Uncontrolled diabetes (HbA1c > 8%, or current use of insulin)
Significant systematic illness or infection in the past 30 days
Pregnant women
Space-occupying lesion in brain that contraindicates LP
Imaging within one year prior to enrollment that identifies any exclusionary lesions

Supplementary Table 1 | Eligibility criteria

DOMAIN	MEASURE	COMPONENT TASKS/SUBTESTS
Overall cognition	Mini Mental State Exam (MMSE)	Global cognition
Intelligence	Hopkins Adult Reading Test	Estimates of premorbid intelligence
Attention	WAIS – Digit Span	Simple aural attention and working memory
Cognitive speed	WAIS – Digit Symbol Substitution Test	Timed coding
Language	Verbal Fluency<br>Boston Naming Test – 30	Timed letter-cued and category-cued word generation<br>Confrontation naming
Episodic memory	Free and Cued Selective Reminding Test	Immediate and delayed recall
Verbal memory	Hopkins Verbal Learning Test-Revised<br>WMS – Logical Memory	Verbal list learning, recall, and recognition<br>Learning, recall, and recognition of stories
Visual memory	Brief Visual Memory Test – Revised	Learning and recall of simple geometric figures
Executive function	Trail Making Test (A & B)	Visual sequencing and mental flexibility
Mood	Geriatric Depression Scale	Self-reported measures of depression in adults >50
Disease staging	Clinical Dementia Rating Scale	Semi-structured interview designed to assess domains of cognitive and functional performance

Supplementary Table 2 | Neuropsychological battery
